# Effects of combination therapy with Shenfu Injection in critically ill patients with septic shock receiving mechanical ventilation: A multicentric, real-world study

**DOI:** 10.3389/fphar.2022.1041326

**Published:** 2022-11-09

**Authors:** Xiaoqian Li, Fan Huang, Lixia Zhu, Tianyi Luo, Yuzhuo Zhang, Huiwen Gu, Liheng Guo, Shuai Mao

**Affiliations:** ^1^ Second Clinical College, Guangzhou University of Chinese Medicine, Guangzhou, China; ^2^ Department of Critical Care Medicine, Guangdong Provincial Hospital of Chinese Medicine, Guangzhou, China; ^3^ Guangdong Provincial Branch of National Clinical Research Centre for Chinese Medicine Cardiology, Guangzhou, China; ^4^ Medical Information Engineering, Guangzhou University of Chinese Medicine, Guangzhou, China

**Keywords:** septic shock, Shenfu injection, combination therapy, mechanical ventilation, critically ill patients, meta-analysis, retrospective study

## Abstract

**Background:** Septic shock has increasingly become a cause of death threatening human survival. Shenfu Injection (SFI), a patented Chinese medicine, has been widely used in the treatment of patients with sepsis and cardiovascular diseases domestically. We sought to examine whether combination therapy with SFI can improve clinical outcomes in critically ill patients undergoing mechanical ventilation (MV).

**Methods:** This real-world, multicenter retrospective trial enrolled consecutive adult patients with sepsis requiring MV from four medical/surgical intensive care units (ICUs) in China between August 2016 and September 2021. Patients were identified from the medical information department database of each center and assigned to either of two groups (SFI or control) on the basis of the initial treatment received. The primary outcome was 28-day all-cause mortality, and the durations of vasopressor therapy and MV, the ICU length of stay, and costs were assessed as secondary outcomes. Subsequently, we performed a meta-analysis of randomized controlled trials (RCTs) on SFI published before July 2021 to verify our conclusions.

**Results:** 2311 mechanically ventilated patients with septic shock (1128 patients in the SFI group and 1183 in the control group) were analyzed. The survival probability during the first 28 days after admission in the SFI group was greater than that in the control group [*p* < 0.01 by log-rank test; hazard ratio (HR), 0.56; 95% confidence interval (CI), 0.39–0.72]. Patients in the SFI group also experienced a significantly reduced duration of vasopressor therapy [7.28 (95% CI, 6.14–8.42) *vs.* 12.06 (95% CI, 10.71–13.41) days, *p* < 0.001], more ventilator-free days [6.49 (95% CI, 5.42–7.55) *vs.* 10.84 (95% CI, 9.59–12.09) days, *p* < 0.001], a shorter ICU length of stay [18.48 (95% CI, 17.59–19.38) *vs.* 23.77 (95% CI, 22.47–25.07) days, *p* < 0.001], and more time free from organ failure [14.23 (95% CI, 12.94–15.52) *vs.* 19.07 (95% CI, 16.09–22.05) days, *p* < 0.001]. No major adverse effects were reported in either group.

**Conclusion:** Among critically ill patients requiring MV, combination therapy with SFI can improve the survival probability without any obvious adverse reactions.

## 1 Introduction

Sepsis is a condition of life-threatening organ dysfunction in which ≥ 1 organs are damaged due to upregulation of the immune response. Septic shock is a severe state of sepsis wherein underlying circulatory and cellular/metabolic abnormalities are profound enough to substantially increase the risk of mortality (Singer et al., 2016). According to an epidemiological survey (Font et al., 2020), the trend of sepsis has been expected to continue, as 1.7 million sepsis cases and nearly 250,000 deaths from sepsis occur in the United States each year, particularly among septic shock patients. Despite the World Health Organization declaring the management of sepsis and septic shock to be a global health priority (Kumar, 2020), the high morbidity and mortality rates in intensive care units (ICUs) still prove that sepsis shock remains a major medical and economic problem worldwide (Fleischmann et al., 2016). Multiple organ dysfunction syndrome, which is driven on by septic shock and an unchecked inflammatory response, is the most dangerous consequence of sepsis. Since the lung is the most severely affected organ in sepsis patients, sepsis-related lung damage is a fearsome complexity that significantly contributes to the high mortality rate. As a result, more than 50% of patients who have severe sepsis or septic shock suffer acute respiratory distress syndrome (Sevransky et al., 2004). Mechanical ventilation (MV), which provides adequate respiratory support and reduces lung injury (Zampieri And Mazza, 2017), is one of the most frequently used life-support measures in septic shock. The need for MV in septic shock patients is a consequence of diverse pathophysiologic conditions, leading to impaired oxygenation and/or ventilation. Whereas, prolonged MV due to failing infection control or hemodynamic disability can result in worse outcomes and a major economic burden (Tobin And Manthous, 2017), meanwhile several studies have reported that sepsis is the mortality-independent risk factor of patients with MV (Epstein And Vuong, 1999; Esteban Et Al., 2002).

Botanical drugs and their derivatives have been used extensively in disease research and treatment for thousands of years. Shenfu Injection (SFI), a classic prescription of traditional Chinese medicine, is widely used for the prevention and treatment of many disorders. It is derived from a famous formula recorded in the *Ji Sheng Fang* by Yonghe Yan during the Song Dynasty (A.D.1253), consisting of 30 g *Aconitum carmichaeli Debeaux* and 15 g *Panax ginseng C.A. Mey*. *Ginsenoside* and *Aconitine* are the main active ingredients in SFI (Liu et al., 2015), and they preform critical roles in stabilizing the hemodynamic status, improving the microcirculation, and regulating cell metabolism. Animal studies have demonstrated that SFI can prevent excessive inflammatory reactions, inhibit mitochondrial apoptosis as well as promote nitric oxide release (Li et al., 2014; Zhang et al., 2016a; Xu et al., 2020). Several clinical studies suggested that SFI is an effective treatment, but them were mostly limited to sample capacity (Li et al., 2015; Li et al., 2016; Zhang et al., 2017a; Fan et al., 2019), combination treatments (Li et al., 2015; Zhang et al., 2017b; Jin et al., 2017; Shao et al., 2020), and some studies used for myocardial diseases (Wei et al., 2015; Guo et al., 2017; Jin et al., 2017; Wang et al., 2019; Wang et al., 2021). To date, no large-scale clinical trials have been performed to evaluate whether SFI combined with routine treatments is effective in septic shock patients receiving MV.

In this study, we used a robust, multicenter database of consecutive septic shock patients who received MV to examine whether combination therapy of SFI is a better treatment option in a real-world setting. Subsequently, we assessed the effectiveness of these outcomes at the trial level *via* a meta-analysis of all adjuvant randomized controlled trials (RCTs) for the management of sepsis patients.

## 2 Materials and methods

### 2.1 Shenfu Injection

China Resources Huarun Sanjiu Medical & Pharmaceutical Co. Ltd. (Ya’an, Sichuan, China), who manufactured SFI (batch number: Z51020664, Z51022664) for this study, is a Good Manufacturing Practice–certified company in China. SFI is an extracted solution derived from *Aconitum carmichaeli Debeaux* (Ranunculaceae; Aconiti Lateralis radix praeparata) and *Panax ginseng C.A. Mey* (Araliaceae; ginseng radix et rhizoma)*. Aconitum carmichaeli Debeaux* has certain toxicity, of which fat-soluble alkaloids and water-soluble alkaloids are its toxic components and toxin-control ingredients, separately, clinically generally decoction or long-term decoction. A flow chart of the SFI preparation process has been described in Supplementary Figure S1. Briefly, the active constituents of them were initially extraction from the two botanical drugs using water precipitation and ethanol reflux, respectively. Then, the two Chinese botanical drug—*Panax ginseng C.A. Mey* and *Aconitum carmichaeli Debeaux* in crude form were filtered and concentrated into 1 mg/ml and 2 mg/ml solution respectively and mixed to form the SFI (specification, 50 ml/bottle; concentration, 0.1 g/ml). After the above preparation process, the toxic fat-soluble alkaloids in the crude *Aconitum carmichaeli Debeaux* liquid are greatly reduced. This technology has obtained a patent certificate issued by the State Intellectual Property Office of China (patent number: ZL96117458.7). Its quality was ensured by the use of Bosch potting system of Germany, Milipore ultrafiltration system of America and automatic lamp inspection system of Japan during production and controlled in compliance with the standard of China Food and Drug Administration (approval number: WS3-B-3427-98-2013). Each SFI was deposited in a sealed, light-protected environment at ambient temperature.

### 2.2 Retrospective study

#### 2.2.1 Study design and data sources

We performed a real-world, retrospective observational study. Information for predicting outcomes with SFI was validated using data from four hospitals and academic institutions in Guangdong Province, China. We followed a retrospectively defined protocol, and ethics committee approval was obtained at participating site (ZE 2021-319-01). Patients were informed that their codified data would be used for the study.

#### 2.2.2 Patients

Adult patients with septic shock who received MV > 24 h after admission between August 2016 and September 2021 were eligible for study inclusion. The identification of patients was based on Sepsis 3.0 (Singer et al., 2016), and the presence of any of the following exclusion criteria resulted in enrollment ineligibility: 1) age of <18 or >80 years; 2) confirmed pregnancy; 3) Those who have neurological diseases or organic brain damage or deep coma; 4) Mentally ill, senile dementia and other persons without capacity for civil conduct; 5) Those with cognitive impairment, hearing impairment and other factors who cannot communicate.

#### 2.2.3 Procedures

We assigned all patients to either the SFI group or the control group. The following information were extracted before intervention: sex, age, body mass index, heart rate, Sequential Organ Failure Assessment score, source of infection, and comorbidities. Two groups of patients were defined on the basis of initial treatment: participants in the control group received standardized treatment only, while those in SFI group received standardized treatment and intravenous SFI (100 ml of normal saline + 50 ml of SFI every 12 h) until the patient is discharged or died. Standardized treatment refers to the Surviving Sepsis Campaign Bundle (Levy et al., 2018) updated in 2018 and included fluid resuscitation, broad-spectrum antibiotics, and standard care. Patients with hypotension or a lactate level ≥ 4 mmol/L were rapidly administered 30 ml/kg of intravenous crystalloid. Monitoring serum lactate level dynamically, if initial lactate was elevated (>2 mmol/L), it would be remeasured within 2–4 h to guide resuscitation to normalize lactate in patients with elevated lactate levels as a marker of tissue hypoperfusion. Empiric broad-spectrum therapy with one or more intravenous antimicrobials was started immediately to cover all likely pathogens. Once pathogen identification and sensitivities are established, empiric antimicrobial therapy was narrowed. Vasopressors were given if the patient was hypotensive during or after fluid resuscitation to maintain a MAP of ≥ 65 mmHg. Symptomatic treatment was administered in other cases until the patient was discharged or died. The MV time in both groups was >24 h. Available reports of adverse events were collected from the medical files of patients.

#### 2.2.4 Outcomes

Clinical response after treatment initiation was retrospectively assessed by the investigators using data obtained from the medical charts. The primary study outcome was 28-day all-cause mortality, while the duration of vasopressor therapy, median time to extubation, ICU length of stay, time free from organ failure, and costs were assessed as secondary outcomes. Time to extubation was defined as the time from start of the ventilator to withdrawal of the ventilator.

### 2.3 Meta-analysis

To further evaluate the therapeutic effect of SFI on sepsis, we conducted a systematic evaluation, which can provide useful information and promote an in-depth understanding of the mechanism. PubMed, China National Knowledge Infrastructure Database, VIP Chinese Science and Technology Journal Database and Wanfang Data were searched to find relevant article on the efficacy of SFI on patients with sepsis by two reviewers independently. The search period is for each database to be established until June 2021 using the following search keywords: sepsis, Shen-Fu. Additionally, we only considered articles of which entire texts could be retrieved for data analysis. Then, two reviewers independently extracted data from the included studies onto a standard form. Search strategies was included in the Supplementary Materials available online.

### 2.4 Statistical analysis

Continuous data are described using mean (SD) or median (IQR), and categorical data are described using frequencies and proportions (%). All continuous variables were tested for distribution normality with Kolmogorov-Smirnov test, then compared using the Wilcoxon rank-sum test, and Pearson’s chi-squared test or Fisher’s exact test was used for comparisons of categorical variables between the two study groups. Log-rank statistics were used to assess the effects of SFI on 28-day mortality, durations of vasopressor and ventilator therapy, ICU length of stay, and time free from organ failure, with hazard ratios (HRs) and 95% confidence interval (CIs) estimated using a Cox proportional hazards model. We constructed three models: model 1, no covariates were adjusted; model 2, only adjusted for sociodemographic data; and model 3, adjusted for all covariates. The time-to-event analyses were conducted used Kaplan–Meier survival curves. All analyses were completed using the SPSS statistical software, version 22.0 (IBM Corporation, Armonk, NY, United States). Two-sided *p* values of <0.05 were considered to indicate statistical significance.

## 3 Results

### 3.1 Retrospective study

#### 3.1.1 Population description

**FIGURE 1:**
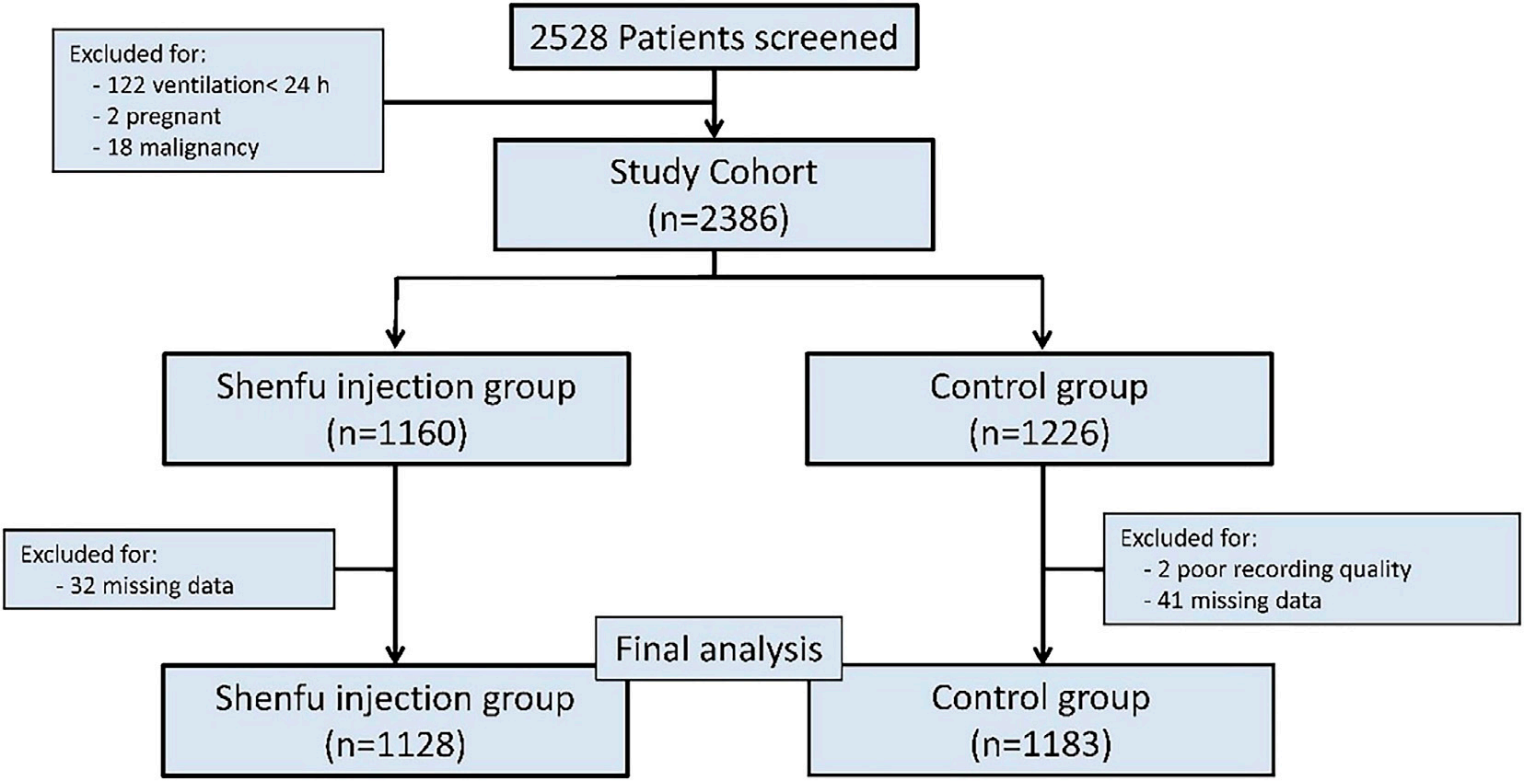
Study profile: Study profile for patients included in this study, and data cutoff was September 2021.

During the review time frame, 2528 patients were diagnosed with septic shock and treated with MV. Among them, 217 patients were excluded. In the end, a total of 2311 patients were enrolled in this study, including 1128 in the SFI group and 1183 in the control group (Figure 1).

#### 3.1.2 Baseline characteristics

The two study groups were comparable at baseline with respect to demographics, severity of illness, comorbid conditions, and ICU admission diagnoses (Table 1).

**TABLE 1:** Baseline demographics of patients.

	Shenfu injection group (n = 1128)	Control group (n = 1183)	*p*-Value
Age, y	71 [60, 81]	70.0 [59, 80]	0.18
Men, No. (%)	682 (60.50)	743 (62.80)	0.26
BMI, kg/m2	27 [25, 29]	26 [24, 29]	0.60
SOFA at enrollment	9 [6, 11]	9 [6, 10]	0.78
Source of infection, No. (%)			
Lung	479 (42.46)	496 (41.93)	0.82
Abdominal	246 (21.81)	223 (18.85)	0.09
Urogenital	109 (9.66)	125 (10.57)	0.52
Systolic blood pressure (mmHg)	78.73 [65.13, 96.48]	76.21 [67.11, 94.38]	0.69
Central venous pressure (mmHg)	8.30 [6.50, 11.20]	9.40 [6.50, 12.90]	0.42
Heart rate (beats/min)	116.20 [81.20, 139.30]	122.40 [88.20, 144.60]	0.32
Lactates (mmol/L)	5.35 [1.12, 10.98]	5.12 [1.58, 11.54]	0.19
PaO2/FiO2	178.30 [111.20, 321.20]	182.10 [124.30, 367.30]	0.88
ALT, U/L	11.18 [0.13, 26.80]	13.06 [0.34, 26.33]	0.99
Creatinine, μmol/L	73.22 [11.19, 116.50]	69.21 [8.92, 106.92]	0.12
eGFR, ml/min • 1.73m2	69.08 [33.47, 91.98]	66.48 [38.33, 95.66]	0.42
PT, s	15.51 [12.13, 16.24]	15.34 [12.05, 17.45]	0.19
TBIL, μmol/L	20.41 [6.47, 36.61]	20.69 [6.57, 37.09]	0.89
Comorbidities, No. (%)			
Diabetes	234 (20.74)	259 (21.89)	0.53
Hypertension	463 (41.04)	516 (43.62)	0.23
Coronary disease	198 (17.55)	212 (17.92)	0.86
Chronic kidney disease	102 (9.04)	94 (7.94)	0.38
COPD	321 (28.45)	309 (26.12)	0.23

#### 3.1.3 Primary outcome

**FIGURE 2:**
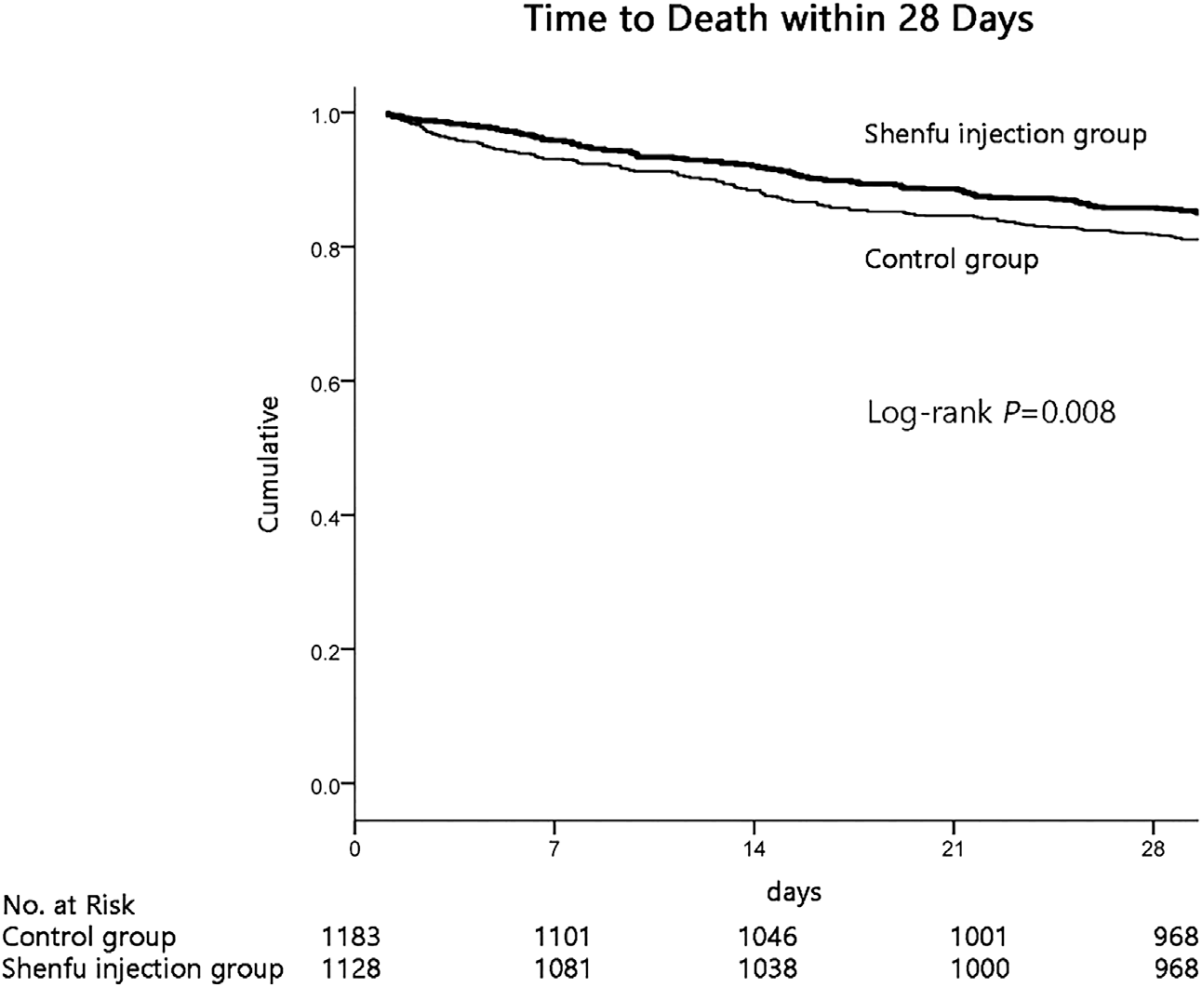
Kaplan-Meier graph of 28 days survival probability in two group.

Importantly, the results demonstrated that the survival probability during the first 28 days after admission in the SFI group was greater than that in the control group (84.8% *vs.* 80.7%; *p* = 0.008 by log-rank test; Figure 2). Cox proportional hazards model was used to adjust confounding factors, with the effect of baseline clinical and demographic characteristics (Table 1) was considering, this difference persisted (HR, 0.749; 95% CI, 0.613–0.914, *p* = 0.004; Table 2).

**TABLE 2:** Effects of SFI in septic shock patients receiving MV in different models.

Variable	Crude model		Model 1		Model 2	
	HR (95%CI)	*p*-value	HR (95%CI)	*p*-value	HR (95%CI)	*p*-value
28-day mortality	0.766 (0.628–0.933)	0.008	0.752 (0.617–0.917)	0.005	0.749 (0.613–0.914)	0.004
vasopressor therapy	0.705 (0.641–0.776)	*p* < 0.001	0.701 (0.637–0.771)	*p* < 0.001	0.704 (0.639–0.775)	*p* < 0.001
length of ICU stay	0.734 (0.668–0.808)	*p* < 0.001	0.729 (0.663–0.802)	*p* < 0.001	0.732 (0.665–0.806)	*p* < 0.001
time to extubation	0.692 (0.630–0.761)	*p* < 0.001	0.687 (0.625–0.756)	*p* < 0.001	0.687 (0.625–0.756)	*p* < 0.001
organ failure	0.814 (0.745–0.889)	*p* < 0.001	0.807 (0.738–0.882)	*p* < 0.001	0.819 (0.749–0.896)	*p* < 0.001

Model 1: adjusted for sex and age.

Model 2: adjusted for sex, age, body mass index, heart rate, blood creatinine, glomerular filtration rate, brain natriuretic peptide, cardiac troponin, creatine kinase isoenzymes, procalcitonin, total bilirubin and prothrombin time.

#### 3.1.4 Secondary outcomes

**FIGURE 3:**
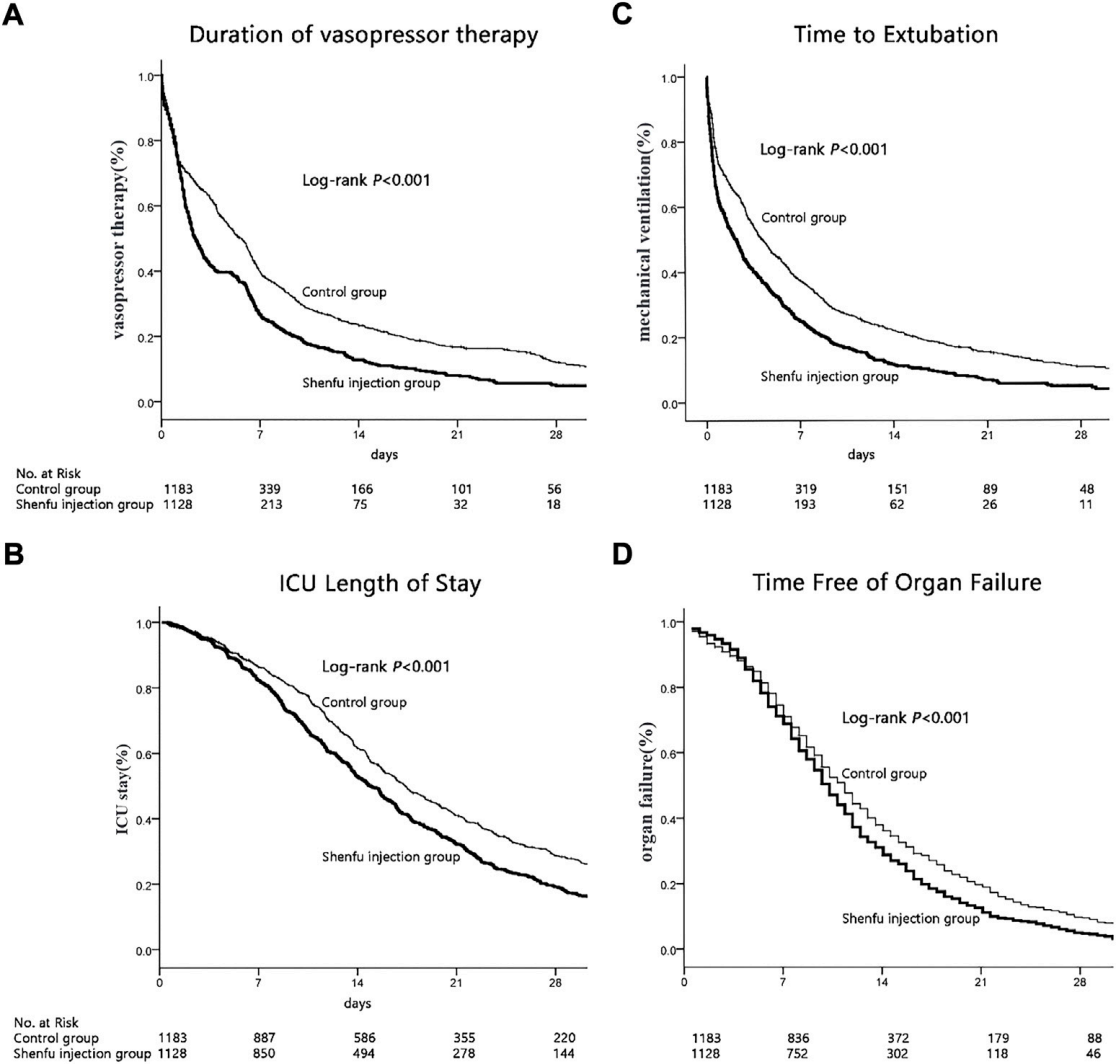
Secondary outcomes. **(A)**Kaplan-Meier graph of vasopressor therapy. **(B)**Kaplan-Meier graph of ICU stay. **(C)**Kaplan-Meier graph of mechanical ventilation. **(D)**Kaplan-Meier graph of organ failure.

The median time of vasopressor therapy was 4.78 days shorter in the SFI group than the control group [7.28 (95% CI, 6.14–8.42) *vs.* 12.06 (95% CI, 10.71–13.41) days, *p* < 0.001, by log-rank test; Figure 3]. Cox proportional hazards model showed that this difference persisted after adjust confounding factors (HR, 0.704; 95% CI, 0.639–0.775, *p* < 0.001; Table 2).

The median length of ICU stay in the SFI group was also shorter than that in the control group [18.48 (95% CI, 17.59–19.38) *vs.* 23.77 (95% CI, 22.47–25.07) days, *p* < 0.001, by log-rank test; Figure 3]. Cox proportional hazards model showed that this difference persisted after adjust confounding factors [HR, 0.732; 95% CI, 0.665–0.806, *p* < 0.001; Table 2].

The median time to extubation was 1.2 days shorter in the SFI group than the control group [6.49 (95% CI, 5.42–7.55) *vs.* 10.84 (95% CI, 9.59–12.09), *p* < 0.001 by log-rank; Figure 3]. Cox proportional hazards model showed that this difference persisted after adjust confounding factors (HR, 0.687; 95% CI, 0.625-0.756, *p* < 0.001; Table 2).

Moreover, The SFI group also experienced a longer time free from organ failure than the control group [14.23 (95% CI, 12.94–15.52] *vs.* 19.07 (95% CI, 16.09–22.05) days, *p* < 0.001 by log-rank test; Figure 3]. Cox proportional hazards model showed that this difference persisted after adjust confounding factors (HR, 0.819; 95% CI, 0.749-0.896, *p* < 0.001; Table 2).

#### 3.1.5 Costs

**FIGURE 4:**
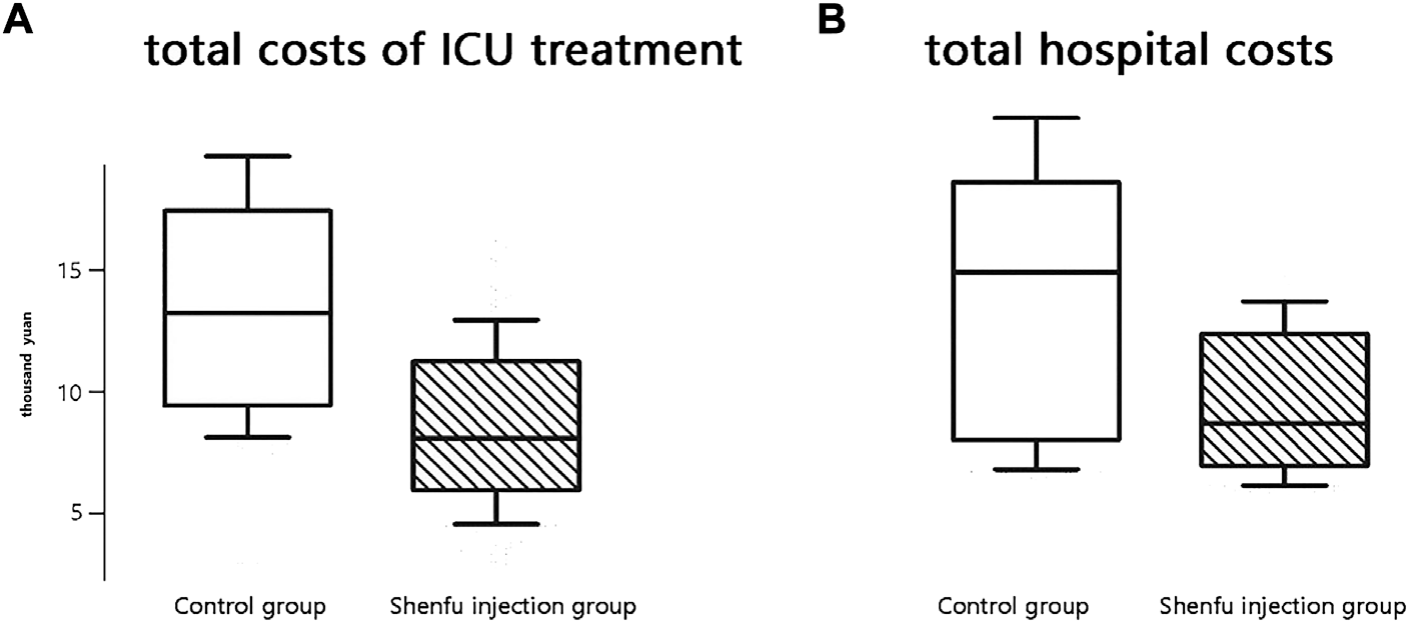
Boxplot of costs in two groups. **(A)**total costs of ICU treatment. **(B)**total hospital costs.

The median cost of ICU treatment was 88341 yuan (95% CI, 76740–112053) in the SFI group compared to 137712 yuan (95% CI, 92516–173201) in the control group (*p* < 0.001). Moreover, the median hospital cost of the SFI group was significantly lower than that of the control group [108150 (95% CI, 64292–134811) *vs.* 153111 (95% CI, 87931–184323) yuan, *p* < 0.001; Figure 4].

#### 3.1.6 Adverse effects

No case of obvious rash, hallucination, headache, thrombophlebitis, bradycardia and tachycardia. Or ataxia occurred in either group.

### 3.2 Meta-analysis

**FIGURE 5:**
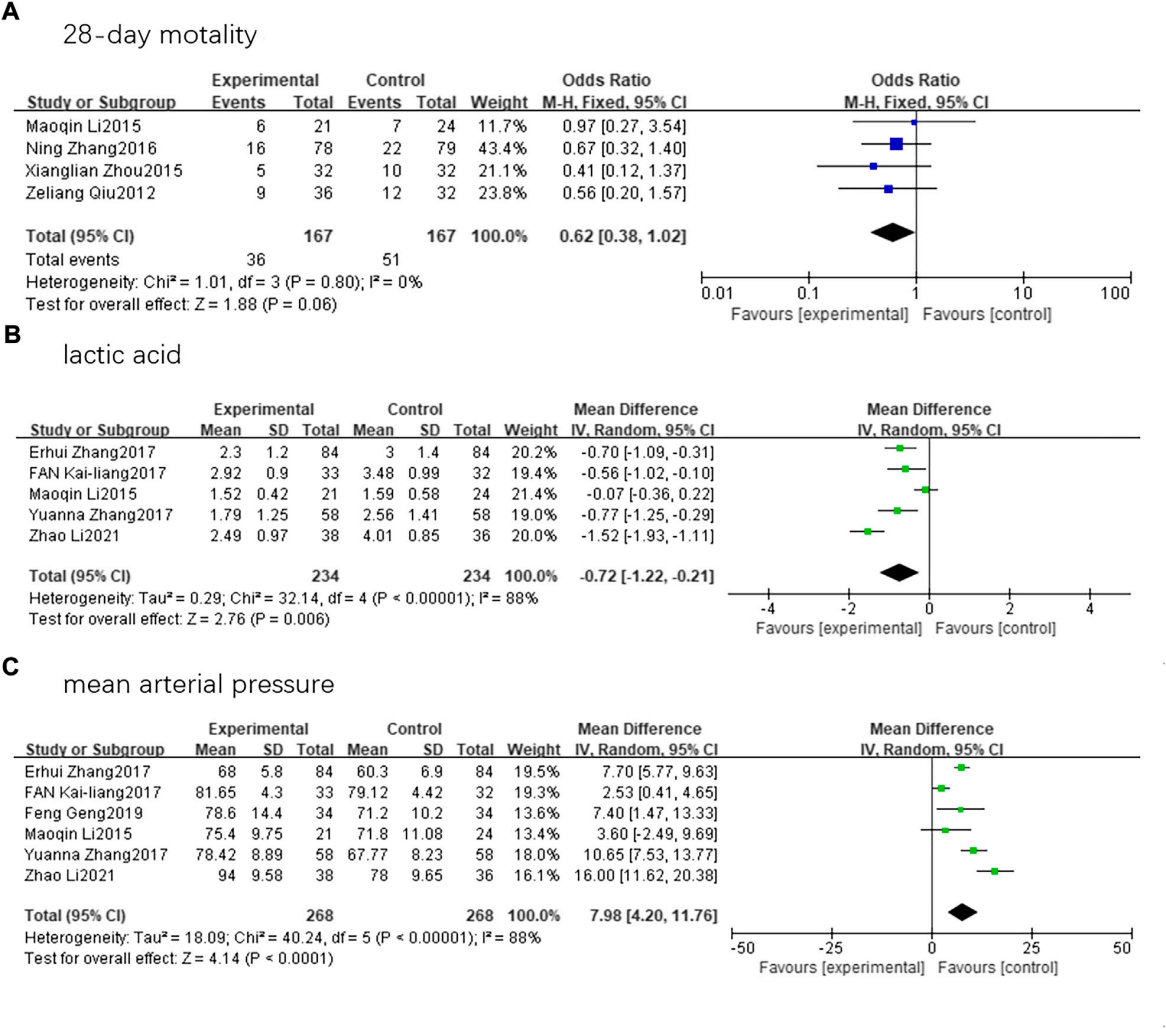
Forest plots. **(A)**28-day mortality. **(B)**lactic acid. **(C)**mean arterial pressure.

Nine randomized controlled trials involving 825 patients (414 in SFI group and 411 in control group) that met the inclusion criteria were finally assessed. Five articles reported on lactic acid levels (Li et al., 2015; Zhang et al., 2017c; Lv et al., 2017; Zhang, 2017; Zhao et al., 2021). We conducted a statistical analysis using a random-effects model and discovered that there was a substantial difference in lactic acid levels between the two groups (mean difference, −0.72; 95% CI, −1.22 to −0.21; Z = 2.78; *p* = 0.006). Six articles reported the MAP (Li et al., 2015; Zhang et al., 2017c; Lv et al., 2017; Zhang, 2017; Geng et al., 2019; Zhao et al., 2021). A random-effects model revealed that patients with septic shock receiving SFI therapy had a significantly improved MAP compared to the control group (mean difference, 7.98; 95% CI, 4.20–11.76; Z = 4.14; *p* < 0.00001). However, the decrease in 28-day mortality (Qiu et al., 2012; Li et al., 2015; Zhou et al., 2015; Zhang et al., 2017a) among patients receiving SFI therapy compared to those in the control group was not statistically significant (odds ratio, 0.62; 95% CI, 0.38–1.02; Z = 1.88; *p* = 0.06) (Figure 5).

## 4 Discussion

Herein, our investigation indicated that SFI could reduce the 28-day all-cause mortality risk and shorten the time to extubation, durations of vasopressor therapy and organ failure, and ICU length of stay to improve the clinical prognosis in a large case series of patients with sepsis shock receiving MV. In addition, we found that SFI could also reduce both ICU and hospital costs. Our meta-analysis results, a significantly desirable outcome from another perspective, confirmed that SFI can decrease the lactic acid level and increase MAP in sepsis patients, but its effect on reducing 28-day mortality is unclear.

One of our key findings is that SFI could reduce the 28-day all-cause mortality risk. A multicenter, prospective randomized study (Li et al., 2016) demonstrated that SFI may improve the 7-day survival in patients with impaired lactate clearance (≥ 4.5 mmol/L), agreeing well with the results archived in a recent meta-analysis (Huang et al., 2019) that included 19 studies covering a total of 1505 patients, which showed that concurrent therapy with SFI for septic shock patients might reduce 28-day mortality in specific subgroups (4.5 mmol/L ≤ mean arterial lactate level < 7 mmol/L). Whereas, when all patients were taken into account, neither study was able to demonstrate an improvement in 28-day survival that was consistent with another clinical trial (Zhang et al., 2017a) which observed no significant difference in the 28-day mortality. Due to the limitation of the sample size and the selection and publication biases of the literature considered for our meta-analysis, we did not further make a subgroup analysis, which may have contributed to the dispute in 28-day mortality. Importantly, complementing the results of the meta-analysis was the observational arm of our retrospective study which provided additional steady evidence that SFI could reduce the 28-day mortality rate of septic shock patients. Additionally, blood lactic acid, a key marker of microcirculatory function and a crucial tool for judging the severity of sepsis, has a positive correlation with disease severity. According to Mikkelsen et al. (2009) early serum lactate level in severe sepsis patients was positively associated with mortality, regardless of clinically obvious organ failure and shock. As for an animal experiment, SFI dramatically reduces blood lactic acid levels in a dose-dependent manner (Xing et al., 2015), subsequently, the beneficial benefits of SFI on lactate clearance were further supported by a RCT (Li et al., 2015), which is congruent with our meta-analysis. SFI was reported to improve the microcirculation may be achieved by increasing the microvascular flow index, perfusion density, and the proportion of perfused vessels in sepsis patients (Jin et al., 2017). Therefore, reducing blood lactic acid levels and 28-day mortality by SFI may be a potential therapeutic target in septic shock patients.

Consistent with the above findings, our research further demonstrated the benefits of short-term in-hospital outcomes, including the median time of vasopressor therapy, extubation time, length of ICU stay, and organ failure time. Firstly, the occurrence of circulation improvement can be used to explain the outcome of a reduced duration of vasopressor therapy. According to a RCT, SFI could improve both cerebral blood flow during cardiopulmonary resuscitation and hemodynamic status without affecting endogenous catecholamine levels (Shao et al., 2020), thus, at least partially by enhancing myocardial contractility, SFI increases cardiac output and blood pressure and improves tissue perfusion (Mou et al., 2015; Fan et al., 2019), reducing the duration of vasopressor therapy. A scant sample research (Li et al., 2015) revealed that SFI in conjunction with early goal-directed therapy could significantly enhance MAP and further decrease dosages of vasoactive drugs, similarly, in a case series of 157 patients by Zhang and colleagues (Zhang et al., 2017a), patients with SFI had substantially reduced vasopressor use compared with patients in the placebo group. The favorable benefits of SFI on MAP were likewise supported by our meta-analysis mentioned above. Secondly, the shorter time of mechanical ventilation may be attributed to improvements in oxygenation and ventilatory performance. Several studies have reported that the combined use of SFI and conventional early goal-directed therapy protocols in critically ill patients can significantly reduce damage to vital organs, and shorten both ventilation times (Li et al., 2015; Zhang et al., 2017b). Subsequently, Lv et al. (2017) discovered that the co-addition of SFI had positive effects on inflammatory mediator clearance, thereby shortening the time of MV and reducing the risks of complications caused by tracheal intubation and tracheotomy which was consistent to what we found in our trial. Thirdly, the reduction of vasopressor therapy time and MV time further resulted in shorten ICU length of stay and organ failure time. The current RCTs did show the beneficial effects of SFI on the duration of vasopressor use, the severity of illness, and MV time which translated into less organ dysfunction and shorter ICU stay in the septic shock patients (Li et al., 2015; Zhang et al., 2017a). Additionally, our research demonstrated that SFI could lower hospital and ICU costs, enhancing patient quality of life and reducing social and financial burdens. Because of the retrospective nature of the study, the conclusions still have certain limitations, although confounding factors are strictly controlled in our methodology and the sample size is large enough. Relevant multicenter prospective study is carrying out to further confirm our conclusions, which will be the focus of our next work.

A previous spectrometric study on SFI showed that its main components could be classified as aconitine, ginsenoside, and nucleoside (Song et al., 2015). One dynamic ingredient of SFI is aconitine, which has various (anti-inflammatory, analgesic, and anti-tumor) pharmacological properties. Aconitine has been proven to improve myocardial cell pulsation frequency and amplitude, enhance myocardial contractility, increase cardiac output, and reduce myocardial oxygen consumption (Hou et al., 2013), thus having a good effect on heart dysfunction caused by sepsis. Another active ingredient is ginsenosides, which exert anti-inflammatory effects by regulating the balance between pro-inflammatory and anti-inflammatory cytokines. The negative regulation of pro-inflammatory cytokine expressions (tumor necrosis factor α, interleukin-1β, and interleukin-6) and enzyme expressions (inducible nitric oxide synthase and cyclooxygenase-2) was identified as the anti-inflammatory mechanism of ginsenosides in M1-polarized macrophages and microglia (Kim et al., 2017; Chen, 2020). The strong immuno-suppressive effects of ginsenoside Rg6 on Toll-like receptor 4–induced systemic inflammatory responses, such as liposaccharide-induced septic shock and cecal ligation and puncture-induced sepsis (Paik et al., 2019), should be mentioned. Additionally, pharmacological investigations have demonstrated the antioxidant, anti-inflammatory, and immuno-modulatory properties of additional minor components like amino acids and nucleosides (Song et al., 2015; Li et al., 2020). According to the results of the above investigations, SFI, which contains aconitine and ginsenosides as its main ingredients, may enhance the circulation and help remove inflammatory mediators. However, these mechanistic data are exploratory, and further studies are needed to test these theories.

SFI, which is based on a classical prescription of traditional Chinese medicine and processed by modern technology, has been widely used in emergency departments and ICUs in China and has shown beneficial effects on shock and organ ischemia/reperfusion injury (Li et al., 2014; Yin et al., 2014; Varon and Varon, 2015; Xu et al., 2020). Our results support the use of SFI as a supplemental treatment for septic shock patients, with several potential cytomolecular processes. First, it is known that SFI can affect mitochondrial functions. Studies have demonstrated that SFI can improve mitochondrial respiratory function and oxygen production by increasing the enzyme activity of left ventricular Na + −K + −ATP and Ca2 + −ATP, promoting energy metabolism and anti-oxidative damage, consequently reducing myocardial dysfunction and shortening ventilation times in patients with septic shock (Ji et al., 2011; Li et al., 2015). An animal study further suggested that SFI could reduce organ damage by protecting the mitochondrial structure of the myocardium in light of the cytomolecular research outlined above. Mitochondrial dysfunction during sepsis, such as abnormal mitochondrial structure, oxidative stress, mitochondrial permeability transition and mitochondrial uncoupling, can affect energy metabolism, leading to tissue hypoxia (Supinski and Callahan, 2006; Lowes et al., 2008; Takasu et al., 2013; Vanasco et al., 2014). Second, SFI can alleviate cell apoptosis. The Shenfu formula can resist myocardial cell apoptosis by inhibiting the protein expressions of Fas, Fas-L, Bcl-2, and Bax, which may slow down the process of sepsis (Yan et al., 2018; Xu et al., 2020). Apoptosis is essential for the selection of immune cell populations and the maintenance of effective immunological responses during sepsis-induced immunosuppression, conversely, excessive apoptosis during an immune response causes a substantial loss of immune cells, which triggers persistent inflammatory reactions (Ayala et al., 2008; Cao et al., 2019). Third, SFI may reduce inflammation. Previous investigators have discovered that SFI can inhibit inflammatory factors (Jin et al., 2017), prevent excessive inflammatory reactions, and suppress immune reactions (Gu et al., 2013; Zhang et al., 2016b; Gu et al., 2016; Zhang et al., 2017a). A clinical report demonstrates that SFI, when used in conjunction with contemporary medicine, can activate bone marrow stem cells and reduce levels of cytokines, including Fas, tumor necrosis factor α, and interleukin-6 (Liu et al., 2015).

As far as we know, this multicenter study is the largest retrospective investigation of SFI therapy in septic shock patients with MV performed to date. We also systematically searched multiple databases and obtained published data, therefore, presenting the most complete evidence base to date and providing more generalizable inferences that SFI could reduce the 28-day all-cause mortality rate, the time to extubation, the ICU length of stay, and both ICU and hospital costs. However, it must be admitted that several limitations exist in this study. First, all included patients were treated in the same Province of China; thus, there may be regional and racial limitations. Second, although we tried to correct for bias, there may still be confounding factors. The effects of other medications in the patient as well as interactions between multiple drugs cannot be completely ruled out. Given the clinical and economic burden of critical illness, an additional large-scale randomized, double-blinded, placebo-controlled clinical trial should be conducted to investigate whether a substantial number of patients could benefit from a SFI treatment strategy.

## 5 Conclusion

This real-world trial shows that combination therapy with SFI, compared to usual treatment, led to improvements in clinical outcomes in critically ill patients, including reductions in the 28-day all-cause mortality rate (the primary outcome), the duration of vasopressor therapy, and the median time to extubation. It also prolonged the time spent free from organ failure and decreased the ICU length of stay, and costs without increased adverse events.

## Data Availability

The raw data supporting the conclusion of this article will be made available by the authors, without undue reservation.

## Abbreviations

SFI, Shenfu Injection; ICU, intensive care units; RCT, randomized controlled trial; HR, azard ratio; CI, confidence interval; MV, mechanical ventilation; MAP, ean arterial pressure.
