# Supplementary material for: Effects of combination therapy with Shenfu Injection in critically ill patients with septic shock receiving mechanical ventilation: A multicentric, real-world study
Source: Front Pharmacol. 2022 Nov 9;13:1041326. doi: 10.3389/fphar.2022.1041326 (PMC9682251; doi:10.3389/fphar.2022.1041326)
Supplement: Supplementary file 1 [file DataSheet1.docx]

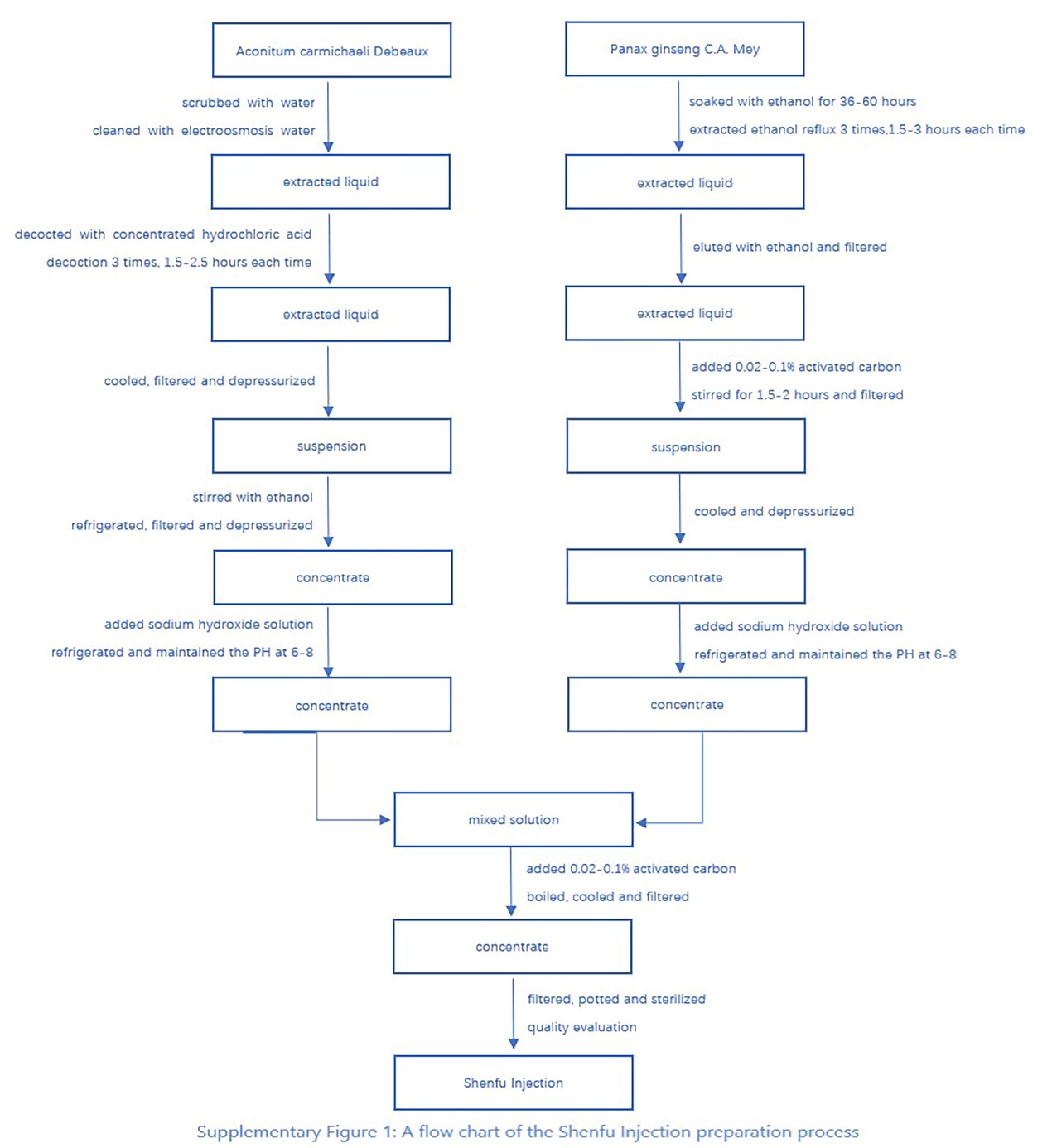


**Search strategies for figure5**

(((((((((((((((((("Sepsis"[Mesh]) OR (Bloodstream Infection[Title/Abstract])) OR (Bloodstream Infections[Title/Abstract])) OR (Infection, Bloodstream[Title/Abstract])) OR (Pyemia[Title/Abstract])) OR (Pyemias[Title/Abstract])) OR (Pyohemia[Title/Abstract])) OR (Pyohemias[Title/Abstract])) OR (Pyaemia[Title/Abstract])) OR (Pyaemias[Title/Abstract])) OR (Septicemia[Title/Abstract])) OR (Septicemias[Title/Abstract])) OR (Blood Poisoning[Title/Abstract])) OR (Blood Poisonings[Title/Abstract])) OR (Poisonings, Blood[Title/Abstract])) OR (Poisoning, Blood[Title/Abstract])) OR (Severe Sepsis[Title/Abstract])) OR (Sepsis, Severe[Title/Abstract])) AND ((("Shen-Fu" [Supplementary Concept])) OR (shenfu[Title/Abstract]))
